# Whole-genome sequence of *Pseudomonas yamanorum* OLsAu1 isolated from the edible wild ectomycorrhizal mushroom *Lactarius* sp. section *Deliciosi*

**DOI:** 10.1128/MRA.00843-23

**Published:** 2023-11-14

**Authors:** Rosario Ramírez-Mendoza, Rodolfo Ángeles-Argáiz, Luis Fernando Lozano Aguirre-Beltrán, Juan José Almaraz-Suárez, Diana Hernández-Oaxaca, Ivette Ortiz-Lopez, Jesus Perez-Moreno

**Affiliations:** 1 Edafología, Campus Montecillo, Colegio de Postgraduados, Texcoco, Estado de Mexico, Mexico; 2 Red de Manejo Biotecnológico de Recursos, Instituto de Ecología, Xalapa, Veracruz, Mexico; 3 Centro de Ciencias Genómicas, Universidad Nacional Autónoma de Mexico, Cuernavaca, Morelos, Mexico; 4 Red de Biodiversidad y Sistemática, Instituto de Ecología, Xalapa, Veracruz, Mexico; The University of Arizona, Tucson, Arizona, USA

**Keywords:** *Pseudomonas*, *Lactarius*, holobiont

## Abstract

We announce the genome sequencing, assembly, and annotation of the OLsAu1 strain and its taxonomic assignment to *Pseudomonas yamanorum*. The isolate comes from a wild edible ectomycorrhizal *Lactarius* sp. mushroom in the *Abies* forest. There is information regarding the strain’s ability to promote plant growth, indicating its potential application in forestry.

## ANNOUNCEMENT

Wild mushrooms are a poorly explored resource for obtaining microbial germplasm. However, the recent perception of Dikarya sporomes as holobionts (1–3) has gained attention for their potential as microbial germplasm source, with biotechnological potential in forestry. However, the ecophysiological insight into mushroom microbiome is still in its infancy. Despite the whole body of information related to the benefits provided by ectomycorrhizal fungi to their associated host plants (4), the perception of mutualistic mushrooms as holobionts has given rise to the notion that some of these benefits might be actually related to their associated microbiome.

For these reasons, we isolated bacteria from a wild edible ectomycorrhizal mushroom, *Lactarius* sp. section *Deliciosi*, which was collected under *Abies religiosa* at Monte Tlaloc, Texcoco, Mexico State, Mexico (19.4194 °N, −98.7369 °W, October 2011). A small fragment of mushroom inner flesh was placed in sterile water and macerated. The strain isolation was carried out by serial dilution, sowing in Pikovskaya agar plates and re-seeded by streaking until isolation. Strains were kept in sterile water at 4°C (5). Isolates have been subject to research focused on their ability to promote plant growth (5). The OLsAu1 strain was selected for whole-genome sequencing because it shows plant growth promotion (5). This strain was previously identified as *Pseudomonas azotoformans* by rRNA 16S phylogeny (MF598585).

A single colony of the OLsAu1 strain was subcultured on solid medium nutrient agar and was grown at 28°C for 2 days. The DNA was extracted using the cetyl trimethyl ammonium bromide (CTAB) method (6). Library construction was performed with the Illumina DNA Prep Kit (Illumina Inc.) following the manufacturer’s instruction. Whole-genome sequencing was performed using the Illumina MiSeq platform (2 × 150 bp) at the “Instituto de Ecología” of the “Universidad Nacional Autónoma de México.”

Raw sequencing data were inspected for quality/quantity with FastQC v0.11.9 (7) and cleaned with TrimGalore v0.6.2 (8) (Table 1). Clean reads were assembled with SPAdes v3.15.3 (9), completeness was assessed with BUSCO v5.3.2 (10), sequencing coverage was calculated with Bowtie v2.3.5.1 (11), and other genome descriptors were done with Quast v5.0.2 (12) and local scripts (Table 1). Functional genome annotations were performed at home with Prokka v1.14.6 (13) and EggNog emapper-2.1.9 online server (14) and also with PGAP v6.6 by NCBI (Table 1). Taxonomic assignment was performed by average nucleotide identity (ANI) analysis with pyani v0.2.11 (15), comparing *Pseudomonas* species of high 16S marker identity with OLsAu1 strain in online BLAST. Default parameters were used for all software.

**TABLE 1:** Genomic assembly features and sequencing^*a*^

Feature	Value
Total DNA amount for sequencing (ng/30 µL)	100
Sequencing reads (×2)	2,137,703
Overall alignment rate (%)	99.33
Sequencing length (bases)	151
Sequencing coverage	93.15
Genome length (≥1,000 bp) (Mbp)	6.79
Genome length (≥0 bp) (Mbp)	6.88
Contigs (≥1,000 bp)	90
Contigs (≥0 bp)	624
Largest contig (Kbp)	774.295
N50 (Kbp)	139.126
L50	14
GC (%)	60.64
Completeness (%)	99.9
Duplications (%)	0
Features (Prokka)	6,254
CDS (Prokka)	6,188
Genes (PGAP RefSeq)	6,300
Proteins (Prokka)	6,188
Proteins (EggNog)	6,407
Protein coding (PGAP RefSeq)	6,171
Non-coding (PGAP RefSeq)	69
Hypothetical proteins (Prokka)	2,353
Hypothetical proteins (EggNog)	518
COGs (Prokka)	2,555
COGs (EggNog)	5,724
KOs (EggNog)	3,697
CAZy (EggNog)	50
PFAM (EggNog)	5,628
EC (Prokka)	1,962
Genome density (genes/Mbp)	930.67

^*a*^ Genome size and contig number include contigs ≥1 Kbp. Sequencing coverage were calculated with Bowtie2. Completeness and duplication values came from BUSCO (*n* = 782).

**Fig 1:**
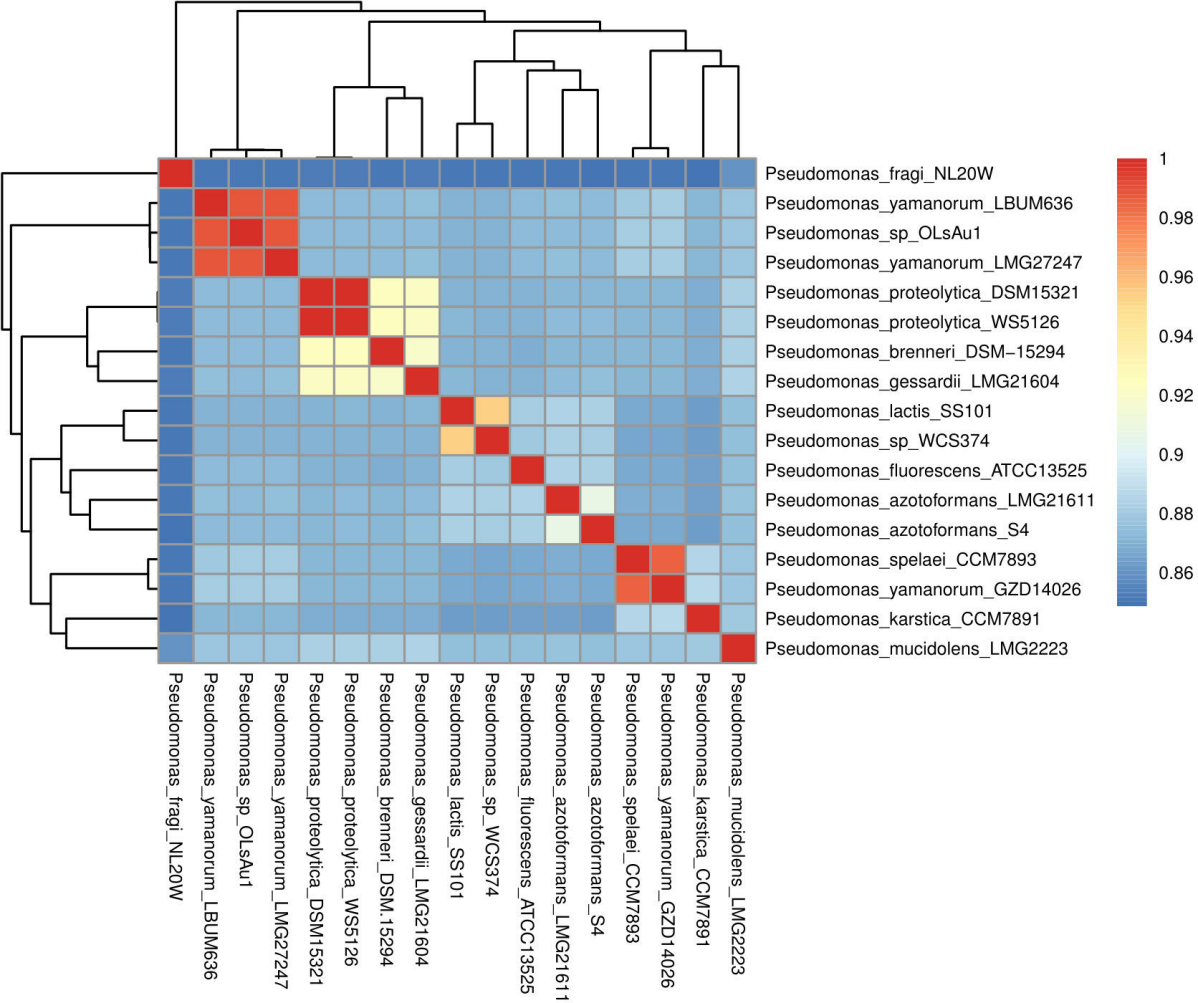
Average nucleotide identity analysis of the genomes of 16 close-relative *Pseudomonas* species with high 16S rRNA similarity to OLsAu1. Colors represent the percentage of identity. Lateral and upper clustering strains were grouped by percentage of identity.

ANI analysis updated the specific assignment to *Pseudomonas yamanorum* (ANI = 0.99) (Fig. 1), a psychrotolerant bacteria originally isolated from the subantarctic environment in southern Argentina (16). The OLsAu1 strain genome (JAVGXC000000000) was assembled to 90 contigs and reached 99.9% completeness and 6.79 Mbp length (Table 1). The OLsAu1 genome assembly matches the analyzed conspecific genome sizes, CG%, and genetic numbers (LBUM636: 6.86 Mbp, 60.6%, 6,140 CDSs; LMG27247: 7.09 Mbp, 60.3%, 6,279 CDSs) (16–18). We do not know of other bacteria isolated from an edible ectomycorrhizal mushroom whose whole genome has been sequenced.

## Data Availability

This Whole Genome Shotgun project has been deposited at DDBJ/ENA/GenBank under the accession number JAVGXC000000000. The version described in this article is version JAVGXC010000000. The assemblies are available under accession number ASM3122489v1. PGAP annotations are available at NCBI in .gb and .sqn. The sequencing libraries are available in SRA under accession number SRX21482516. The BioProject and BioSample accessions are PRJNA1009146 and SAMN37147730. All codes used and Prokka and EggNog annotations are publicly available on the GitHub repository (https://github.com/Rodolfo47/Pseudomonas_sp.OLsAu1.git).
